# Antibiotic use in surgical units of selected hospitals in Ghana: a multi-centre point prevalence survey

**DOI:** 10.1186/s12889-019-7162-x

**Published:** 2019-06-21

**Authors:** Antoinette A. A. Bediako-Bowan, Enid Owusu, Appiah-Korang Labi, Noah Obeng-Nkrumah, Gifty Sunkwa-Mills, Stephanie Bjerrum, Japheth Awuletey Opintan, Cynthia Bannerman, Kåre Mølbak, Jørgen Anders Lindholm Kurtzhals, Mercy Jemima Newman

**Affiliations:** 1 Department of Surgery, School of Medicine and Dentistry, College of Health Sciences, University of Ghana, Accra, Ghana; 2 Department of Surgery, Korle Bu Teaching Hospital, Accra, Ghana; 3 Department of Veterinary and Animal Science, University of Copenhagen, Copenhagen, Denmark; 4 Division of Infectious Disease Preparedness, Statens Serum Institut, Copenhagen, Denmark; 5 Department of Medical Laboratory Sciences, School of Biomedical and Allied Health Sciences, College of Health Sciences, University of Ghana, Accra, Ghana; 6 Department of Microbiology, Korle Bu Teaching Hospital, P.O. Box 77, Accra, Ghana; 7 Department of Clinical Microbiology, Copenhagen University Hospital (Righospitalet), Copenhagen, Denmark; 8 Centre for Medical Parasitology, Department of Immunology and Microbiology, University of Copenhagen, Copenhagen, Denmark; 9 Department of Medical Microbiology, School of Biomedical and Allied Health Sciences, College of Health Sciences, University of Ghana, Accra, Ghana; 10 Department of Public Health, Global Health Section, University of Copenhagen, Copenhagen, Denmark; 11 Formerly Institutional Care Division, Ghana Health Service, Accra, Ghana; 12 Discipline of Community Health, Accra College of Medicine, P. O. Box CT 9828, Cantonments, Accra, Ghana

**Keywords:** Antibiotic use, Surgery, Surgical prophylaxis, Ghana

## Abstract

**Background:**

Improper use of antibiotics leads to the emergence of resistant microorganisms as well as drug toxicity, increased healthcare costs, morbidity and mortality. Globally, an estimated 25–68% of hospitalized patients receive suboptimal antibiotic regimes. Information on the extent of this problem in Ghana is currently limited, particularly in surgical units. To strategize for interventions, we estimated the antibiotic use prevalence in surgical departments in a country-wide point prevalence survey (PPS) in Ghana.

**Methods:**

Between October 2016 and December 2016, we conducted a cross-sectional multi-center country-wide PPS. This involved an audit of in-patients’ records from all units/departments of ten systematically selected hospitals in Ghana. Data were collected with a standardized questionnaire, adopted from the European Centre for Disease Prevention and Control. In this report, we present data on antibiotic use from the surgical units.

**Results:**

Of 2107 eligible patients included in the PPS, 540 patients were identified in surgical units, of which 70.7% (382/540) received antibiotic therapy. A total of 636 antibiotic prescriptions were issued to these surgical patients; 224 (58.6%) for treatment, including 50 for treatment of hospital-acquired infections, and 144 (37.7%) for prophylaxis (medical and surgical). Median duration of antibiotic therapy prior to the survey was 5 days (interquartile range (IQR): 3-8 days). Surgical prophylaxis was administered for longer than the recommended one day in 107 of 144 (88.4%) patients. The choice of antibiotics was largely similar for community- and hospital-acquired infections as well as for prophylaxis. Only 3.7% of patients had microbiological analysis done on clinical samples.

**Conclusion:**

We found a high prevalence of antibiotic use, with the choice of antibiotics, in some cases, inconsistent with the country’s treatment guidelines. Antibiotics were administered for long duration including antibiotics for prophylactic purposes and the majority was started without supporting microbiological analysis. Prescription practices that encourage rational use of antibiotics guided by microbiology and enforcement of antibiotic policy guidelines should be the target for future interventions.

**Electronic supplementary material:**

The online version of this article (10.1186/s12889-019-7162-x) contains supplementary material, which is available to authorized users.

## Background

Correct use of antimicrobials, including antibiotics, can be defined as “the cost-effective use of antimicrobials which maximizes clinical therapeutic effect, while minimizing both drug-related toxicity and the development of antimicrobial resistance” [1]. A recent systematic review unambiguously concluded that “interventions to improve antibiotic prescribing to hospital inpatients” are effective in reducing usage without increasing mortality and with resulting reduction in length-of-stay and healthcare costs [2]. However, this was almost entirely based on studies from North America and Europe and not a single study from Africa was included. Another review, focusing on low- and middle-income countries (LMIC), identified this field as a major research priority to provide tools to curb the spread of antibiotic resistance [3]. In LMIC the situation is further complicated by the fact that lack of access to relevant antibiotics can co-exist with excessive and inappropriate use [4]. Thus, research from high-income countries is of limited value to guide tailoring of antibiotic stewardship programs in African countries.

It is generally thought that inappropriate use of antimicrobials in healthcare facilities is widespread in LMIC but there are relatively few studies in limited geographical areas to document this. Generally, antimicrobial prescribing in hospitals, is said to be suboptimal in 25–68% of cases [3, 5, 6], and in an Australian study, 47% of antibiotic use was found to be discordant with guidelines or microbiological results [7]. Some reasons ascribed to inappropriate antibiotic use include lack of regulation in antibiotic use, high rate of self-medication and lack of treatment compliance as well as lack of access to appropriate and accurate diagnostic facilities [8]. Prescribers’ limited knowledge regarding optimal diagnostic approaches, lack of resources and opportunity for patient follow-up and monitoring, lack of experience and diagnostic uncertainty also lead to increasing episodes of prescribing errors [9, 10].

The present study aims to estimate the prevalence of antibiotic use in surgical departments at all levels of the healthcare system in Ghana and examine its variation according to patient-related and care-related characteristics. The prevalence of multidrug resistant bacteria is widespread in Ghana, higher in the regional hospitals than in the teaching hospitals [11], and a recent study has demonstrated the need for actions aimed at improving antibiotic use [12]. Most literature on antibiotic prescribing practices in surgical units in Africa focuses on surgical prophylaxis and there is a need for comprehensive data on antibiotic use in these settings. Such data are essential to design antibiotic stewardship programs, in Ghana and similar middle-income countries.

## Methods

### Study design

We analyzed a recent cross-sectional multi-center country-wide point prevalence study (PPS) of hospital acquired infections conducted at selected hospitals under the Ghana Health Service/Ministry of Health between September and December 2016 [13]. The PPS was realized through a collaboration between the selected hospitals and the HAI Ghana project research team, an international multicenter and interdisciplinary research network of Ghanaian and Danish experts in the fields of clinical microbiology, interventional studies, cost analysis and ethnographic research. Data on antibiotic use in patients admitted for a surgical specialty were analyzed for this report.

Overall in Ghana, there are 3 teaching hospitals, 10 regional hospitals and 162 district hospitals in the public health sector, with a total bed capacity of 12,806 and a 60.4% occupancy rate. District hospitals form the first referral point from health centers, with a 50–60 bed capacity and facilities for primary clinical care. Regional hospitals form the secondary level for healthcare, providing specialized care and with a 150–200 bed capacity. Tertiary hospitals provide complex tertiary care with availability of varied surgical specialties, a bed capacity ranging from 1000 to 2000 [14].

Ten representative hospitals (2 teaching hospitals, 3 regional hospitals and 5 district hospitals), one from each region, were selected across Ghana using a systematic sampling design adopted from the European Centre for Disease Prevention and Control (ECDC) methodology [13, 15]. The total bed capacity of these 10 hospitals was 4208, representing 32.9% of the total bed capacity for the public hospitals in Ghana.

### Patient selection

We included all patients on admission for a surgical specialty, irrespective of the ward they were admitted on, in the selected hospitals before 8 a.m. on the day of the survey and who had not been discharged from the ward at the time of the survey. We excluded patients admitted after 8 a.m., patients undergoing same day treatment or surgery; out-patients including dialysis patients; and patients in the accident and emergency departments, except for wards attached to these departments where patients are monitored for more than 24 h.

### Procedure

The survey was conducted in a single day over a 12-h period for all hospitals, except for hospitals with large bed capacity (> 1000), where two or three days were used. In these large hospitals however, the survey in a designated department or unit was conducted within a 12-h period, that is 8 a.m. to 8 p.m. on a specified date.

Five personnel at each survey site, usually members of the hospital’s quality assurance team or infection control team, were recruited and trained on the data collection tool, to join the HAI Ghana research team in data collection.

Data were collected from available medical and nursing records using a modified version of the ECDC data collection tool [15]. From the records we identified patients on antibiotic therapy on the day of the survey, and recorded treatment duration for the specified agent and the results of microbiological analysis if performed. Patient-based data further included age, sex, surgical discipline, type of surgery and McCabe score [15].

### Statistical analysis

Data was extracted from the multi-center country-wide study [13] and entered into ACCESS (Microsoft Office 2016) database. Data were cleaned and exported to STATA / MP version 15.1 for analysis. Statistical analysis included appropriate descriptive statistics, subpopulation estimations of percentages, interquartile range and frequencies of patients who had been administered antibiotics or not. In all calculations, the subpopulation of 540 surgical patients was analyzed as a random variable of the 2107 originally sampled patients. In addition, the two-stage sample design was taken into account by using the svyset and svy prefix of STATA. The point prevalence of antibiotic use was reported as a percentage of patients on at least one antibiotic over the total number of patients. Pearson’s chi-square tests, as well as univariate and multivariate logistic regression were used to demonstrate association of various characteristics. *P*-values< 0.05 were considered statistically significant.

## Results

### Patient characteristics

In total, 540 patients were admitted to surgical units in the ten selected hospitals surveyed with the following distribution by hospital category: tertiary 47% (254), regional 18.5% (100) and district 34.4% (186). The median age was 39 years (range 3 days- 89 years [Interquartile range (IQR): 20-56 years]) and the male: female ratio was 1.7:1 Admissions to the general surgical units accounted for 71.5% (386) of patients, followed by 11.3% to the orthopedics and trauma units (Table 1). Most patients, 92% (497), were being managed for non-fatal conditions, 6.3% (34) for ultimately fatal conditions and 1.7% (9) for rapidly fatal conditions. Of all admitted patients, 48% (261) had undergone a surgical procedure and 11% (62) had been diagnosed with a healthcare associated infection during the current admission (Table 1).

**Table 1:** Clinical characteristics and antibiotic use pattern in surgery in hospitals in Ghana

Characteristics	Number of surgical patients on admissionn = 540	Number of surgical patients on antibiotics *n* = 382	Prevalence (95% CI) of antibiotic use amongst surgical patients	Odds ratio (95% CI) Univariable analysis	p-value	Odds ratio (95% CI) Multivariable analysis	Number of antibiotic prescriptions *n* = 636 [Number of prescriptions / persons on antibiotic]
*Hospital type*							
District Hospital	186	120	64.5 (56.0–73.1)	1.0			179 [1.49]
Regional Hospital	100	84	84.0 (68.3–99.7)	2.9 (0.8–9.9)	0.08		166 [1.98]
Tertiary Hospital	254	178	70.1 (64.8–75.3)	1.3 (0.8–2.0)	0.2		291 [1.63]
*Surgical specialty*							
Dental (+ MXF) Surgery	13	9	69.2 (52.2–86.3)	1.0			19 [2.1]
Ear, Nose and Throat	8	7	87.5 (50.6–124.4)	3.1 (0.1–95.1)	0.5		10 [1.43]
General Surgery	386	274	71.0 (61.3–80.7)	1.1 (0.4–2.7)	0.8		466 [1.70]
Neurosurgery	24	15	62.5 (62.5–62.5)	0.7 (0.3–1.6)	0.4		26 [1.73]
Ophthalmology	2	0	0.0	–			0 [0.00]
Orthopedics and trauma	61	40	65.6 (62.1–69.1)	0.8 (0.4–1.9)	0.7		52 [1.30]
Pediatric surgery	33	25	75.8 (67.7–83.8)	1.3 (0.6–3.4)	0.4		42 [1.68]
Urology	13	12	92.3 (92.3–92.3)	5.3 (2.3–11.9)	0.001	5.0 (1.5–15.9)	21 [1.75]
*Age group*							
0–12 (Children)	82	62	75.6 (64.7–86.5)	1.0			111 [1.79]
13–17 (Adolescents)	33	21	63.6 (41.2–86.1)	0.6 (0.2–1.5)	0.2		36 [1.71]
18–29 (Young adult)	84	58	69.0 (61.6–76.5)	0.7 (0.4–1.4)	0.3		100 [1.72]
30–39 (Thirties)	75	51	68.0 (49.6–86.4)	0.7 (0.4–1.3)	0.2		80 [1.57]
40–64 (Middle age)	170	121	71.2 (64.0–78.4)	0.8 (0.4–1.5)	0.4		198 [1.64]
> 65 (Old age)	96	69	71.9 (61.7–82.6)	0.8 (0.4–1.9)	0.6		111 [1.61]
*Gender*							
Male	340	243	71.5 (63.1–79.8)	1.0			397 [1.63]
Female	200	139	69.5 (62.1–76.9)	0.9 (0.6–1.3)	0.6		239 [1.72]
*McCabe Score*							
Non-fatal	497	356	71.6 (63.9–79.3)	1.0			594 [1.67]
Rapidly fatal	9	6	66.7 (16.7–116.6)	0.8 (0.1–5.8)	0.8		8 [1.33]
Ultimately fatal	34	20	58.8 (45.6–72.0)	0.6 (0.3–1.2)	0.1		34 [1.70]
*Surgical procedure done*							
No	278	175	63.1 (50.9–75.3)	1.0			293 [1.67]
Yes	262	207	77.6 (72.1–85.0)	2.1 (1.5–3.1)	0.02	2.2 (1.0–4.6)	343 [1.66]
*HAI documented*							
No	478	323	67.6 (59.8–75.4)	1.0			522 [1.62]
Yes	62	59	95.2 (86.8–103.6)	9.4 (1.5–60.3)	0.02	8.9 (1.4–56.5)	114 [1.93]
*Types of infections*							
*BSI*	*4*	*4*	*100.0*				*9 [2.25]*
*Pneumonia*	*7*	*7*	*100.0*				*18 [2.57]*
*SSI*	*36*	*33*	*91.7 (79.5–103.8)*				*61 [1.85]*
*UTI*	*7*	*7*	*100.0*				*11 [1.57]*
*Other*	*8*	*8*	*100.0*				*15 [1.88]*

*MXF* Maxillofacial, McCabe score: Classification of the severity of underlying medical conditions. Non-fatal disease (expected survival at least five years); ultimately fatal disease (expected survival between one and five years); rapidly fatal disease (expected death within one year); *CI* Confidence interval, *HAI* Healthcare associated infection, *BSI* Blood stream infection, *SSI* Surgical site infection, *UTI* Urinary tract infection

The patients had spent a median of 8 days in hospital before the survey date, ranging from < 1 to 203 days (IQR 4 – 18 days). For patients on antibiotic for treatment, the median duration of stay prior to the survey was 9 days (range 1 – 142 days, IQR 5–18 days) and for patients on antibiotic prophylaxis, it was 6 days (range 0 – 103 days, IQR 3–9 days].

### Antibiotic use

Of all the patients, 382 (70.7% (95% CI 63.6–77.9%)) received antibiotics, with an average of 1.66 prescriptions per patient. Among the 382 patients receiving antibiotics, 44.2% (169) were on one antibiotic, 46.6% (178) on two antibiotics, 7.6% (29) on three and 1.6% (6) were on four antibiotics (Additional file 1:Table S1).

Two major factors significantly affected antibiotic drug use in the univariate analysis: (1) Surgical procedure done or not (78.6% vs 63.1%, respectively, *p* = 0.02) and (2) Presence of a healthcare associated infection (HAI) (95.2% vs 67.6 respectively, p = 0.02). Patients admitted for a urological specialty also had a significantly increased prevalence of antibiotic use (92.3%, *p* = 0.01). In contrast, the hospital type, patient age, sex and disease severity were not associated with antibiotic use (Table 1). In the final multivariable analysis, patients who had a surgical procedure, had an HAI or were managed for a urological condition, remained significantly more likely to be prescribed antibiotics (Table 1) – and these factors were independent of each other with odds ratios similar to that obtained in univariable analysis.

### Antimicrobial agents used

**Fig. 1:**
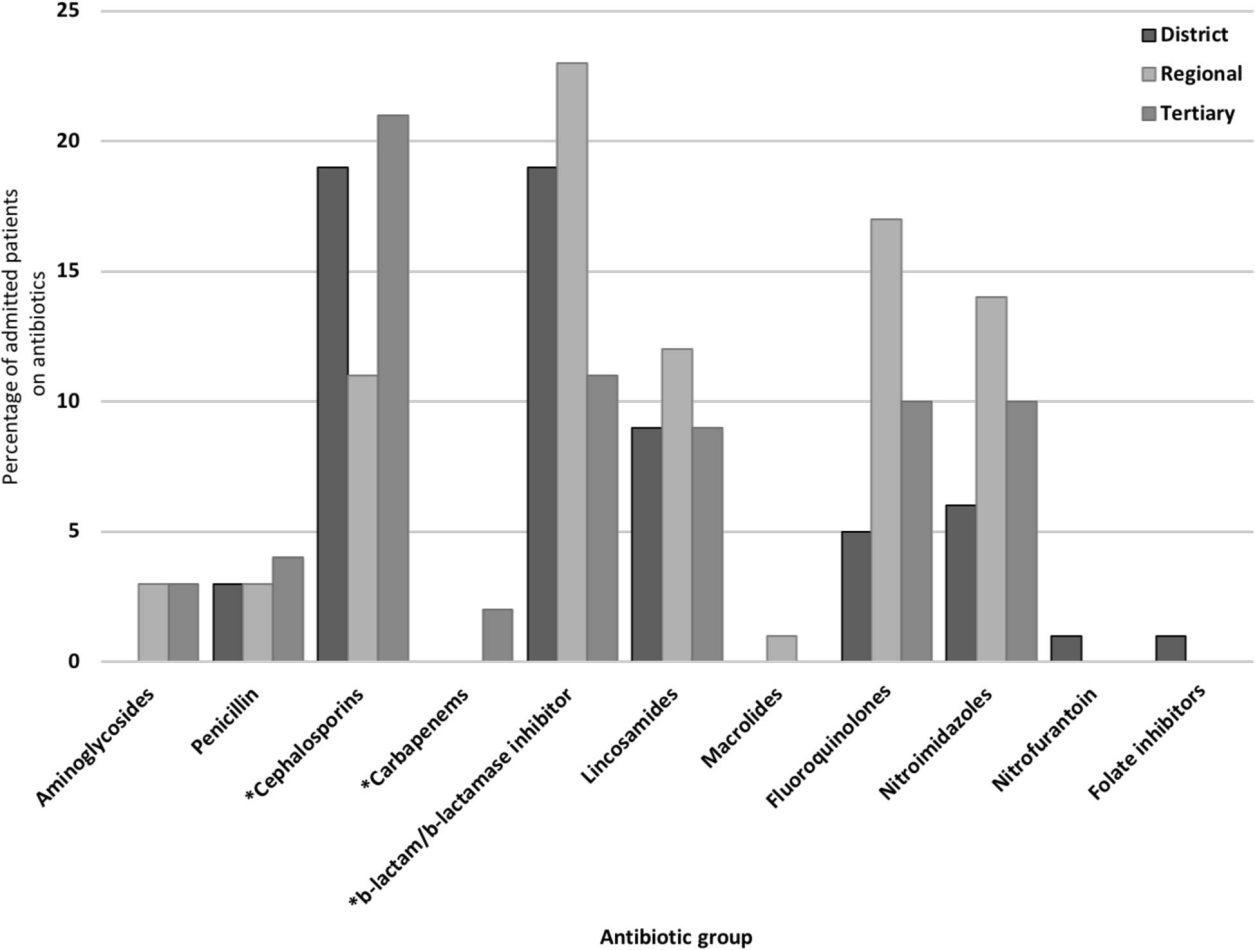
Antibiotic use in different levels of hospitals. The bars show the percentage of patients on antibiotic therapy. Antibiotics are divided in main classes, see text for commonly used drugs in each class. *, significant difference in use of the drug class between levels of healthcare facility (*p* < 0.05)

The most frequently prescribed antibiotics across all levels of facilities were nitroimidazoles (metronidazole) (25.6% (163 of a total of 636 prescriptions)), 2nd and 3rd generations cephalosporins (cefuroxime and ceftriaxone respectively) (20.0% (127)), β-lactam/βlactamase inhibitors (amoxicillin/clavulanic acid) (16.7% (106)), fluoroquinolones (ciprofloxacin and levofloxacin) (12.3% (78)) and lincosamides (clindamycin) (10.2% (65)). The different antibiotic combinations use by groups is shown in Additional file 1: Table S1. There were some differences in the type of antibiotics prescribed between the different levels of hospitals (*p* = 0.002): Penicillins, cephalosporins and carbapenems (meropenem) were used more in tertiary level facilities (35.7%, *p* = 0.03; 57.6%, *p* = 0.01 and 100%, p = 0.01, respectively), whereas β-lactam/ β-lactamase inhibitors were used more in district hospitals (40.9%, p = 0.01). The use of nitroimidazoles, fluoroquinolones, lincosamides and penicillins did not differ between hospital levels (Fig. 1).

The most commonly used antibiotics for treatment of both community- and hospital-acquired infections were cephalosporins, amoxicillin-clavulanic acid, clindamycin, ciprofloxacin and metronidazole (Table 2). The same antibiotics were used for surgical prophylaxis although clindamycin and metronidazole were used less for this indication.

**Table 2:** Indication of antibiotic use in patients of selected hospitals in Ghana

	Indication for treatment (*n* = 382)				
Antibiotic group (n)	Community acquired infection (*n* = 174)	Surgical prophylaxis (*n* = 121)	Hospital acquired infection (*n* = 53)	Medical prophylaxis (*n* = 23)	Unknown indication (*n* = 14)
Aminoglycosides (10)	5	0	3	1	1
Penicillin (28)	15	8	4	1	0
Cephalosporins (92)	40	35	9	5	3
Carbapenems (5)	1	1	3	0	0
β-lactam/β-lactamase inhibitor (88)	31	34	11	7	5
Lincosamides (51)	34	8	6	1	2
Macrolides (1)	0	0	1	0	0
Fluoroquinolones (53)	18	23	8	3	1
Nitroimidazoles (51)	30	11	5	4	1
Nitrofurantoin (2)	0	1	0	0	1
Folate inhibitors (1)	0	0	0	1	0

### Indication for antibiotic use

Patients were prescribed antibiotics for treatment of infections (58.6% (224/382)) or for prophylaxis (37.7% (144/382)), and for 14 patients there were no recorded reason for antibiotic therapy. Of patients prescribed antibiotics for treatment, 77.7% (174/224) were given for community acquired infections – including cellulitis, osteomyelitis, septicemia, urinary tract infections, pyelonephritis, intracerebral abscesses and peritonitis due to typhoid ileitis with perforation, appendix abscesses and perforated duodenal ulcers; and 22.3% (50/224) for hospital acquired infections. Of the antibiotics used for prophylaxis, 16% (23/144) were given for medical prophylaxis and 84% (121/144) for surgical prophylaxis. The contribution of surgical prophylaxis to the total antibiotic usage was 32% (121 of 382 patients given antibiotics). For surgical prophylaxis, 88.4% (107/121) of the patients received antibiotics for more than one day, 9.9% (12/121) for one day and 1.6% (2/121) as a single dose. The distribution between antibiotic use for community- and hospital-acquired infections and prophylactic use was similar at district, regional and tertiary facilities (*p* = 0.7).

### Route of administration

Antibiotics were more commonly administered per the parenteral route (54%) than orally (46%). For individual antibiotic agents, nitroimidazoles (metronidazole) were mainly given by the parenteral route (67%), whereas β-lactam/β-lactamase inhibitors (amoxicillin/clavulanic acid) were mainly given orally (53%). All 3rd generation cephalosporins (ceftriaxone) were given parenterally and 2nd generation cephalosporins (cefuroxime) were given both orally and parenterally (Table 3).

**Table 3:** Antibiotic use pattern with respect to route of administration

	Route of administration (Number of patients = 382 (%)	
	Oral *n* = 176 (46.1%)	Parenteral *n* = 206 (53.9%)
Antibiotic group (n)		*p-value = 0.002*
Aminoglycosides (10)	0	10 (100.0)
Penicillin (28)	10 (35.7)	18 (64.5)
Cephalosporins (92)	41 (44.6)	51 (55.4)
Carbapenems (5)	0	5 (100.0)
β-lactam/β-lactamase inhibitor (88)	46 (52.3)	42 (47.7)
Lincosamides (51)	28 (54.9)	23 (45.1)
Macrolides (1)	1 (50.0)	1 (50.0)
Fluoroquinolones (53)	30 (56.6)	23 (43.4)
Nitroimidazoles (51)	17 (33.3)	34 (66.7)
Nitrofurantoin (2)	2 (100.0)	0
Folate inhibitors (1)	1 (100.0)	0
Duration of antibiotic intake / day		
Median (range)	6 (0–89)	4 (1–33)
[IQR]	[3–9]	[2–7]

### Antibiotic use per source of infection

**Fig. 2:**
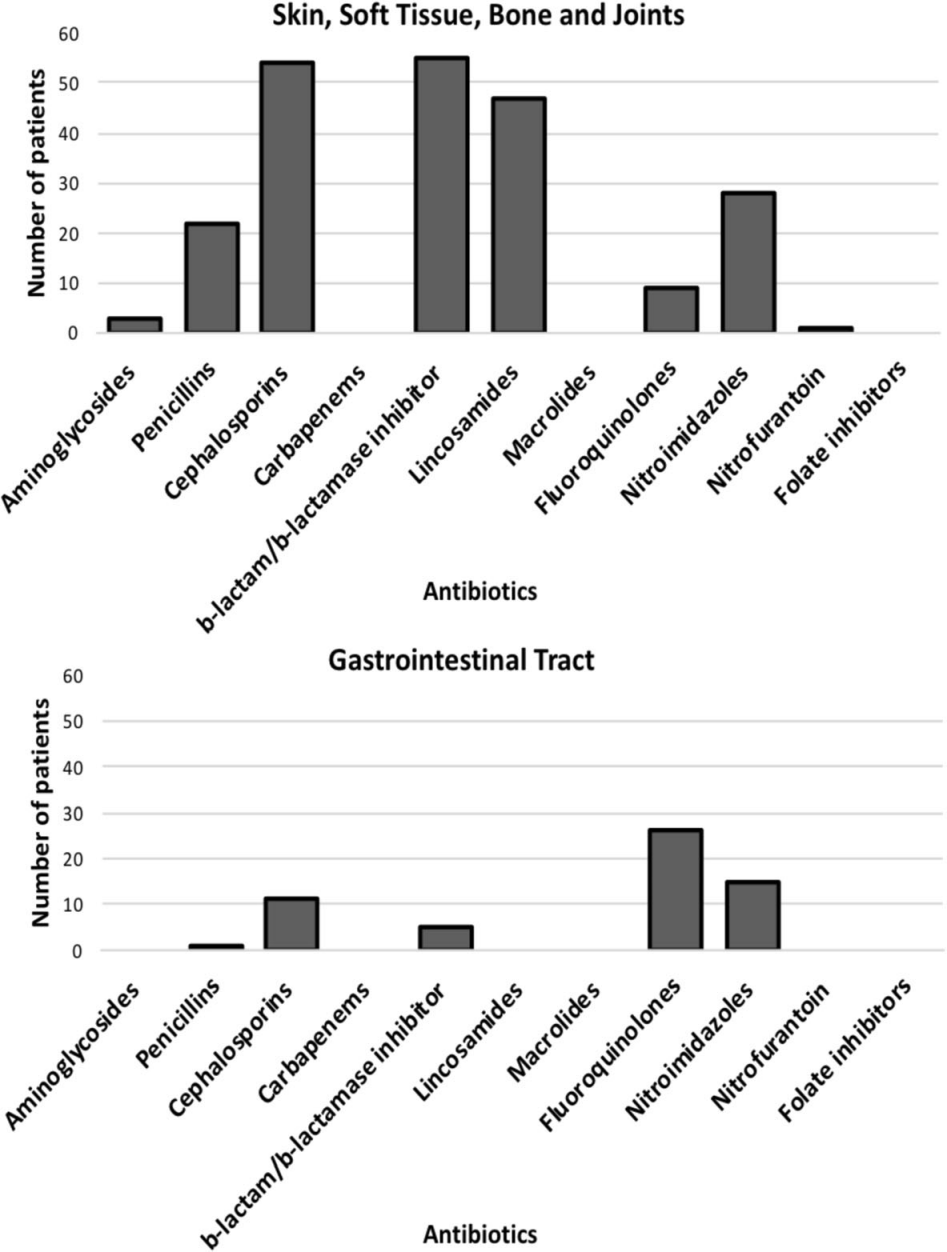
Antibiotic use pattern by organ source of infection. The bars indicate the number of patients being managed with antibiotic for the organ systems: Skin, soft tissue, bone and joints and Gastrointestinal tract. The other organ sources are omitted since they show no clear patterns of antibiotic use due to their small numbers

The most common indications for antibiotic treatment were infections of the skin, soft tissue, bone and joints (SSTBJ) (62.5% (140/224)) and gastrointestinal tract (GIT) (9.8% (22/224)). For prophylaxis, 51.4% (74/144) and 25% (36/144) were given to prevent infections of SSTBJ and GIT infections, respectively. This picture was similar across hospital levels. Of note, cephalosporins (cefuroxime), β-lactam/βlactamase inhibitors, clindamycin and metronidazole use dominated treatment of skin, soft tissue, bone and joint infections whereas fluoroquinolone and metronidazole dominated treatment of gastro-intestinal tract infections (Fig. 2).

### Duration of antibiotic use

The median duration of antibiotic therapy prior to the survey was five days (IQR 3–8 days) with differences across different hospital levels (*p* = 0.02). It was 4 days (range 0-51 days, IQR 2-7 days) in district hospitals, with a wider range of 1–89 days (median 5 days, IQR: 3–8 days) in tertiary level hospitals.

Depending on the indication, median duration of therapy varied from 6 days (IQR 3–9 days) for hospital acquired infections to 1 day for surgical prophylaxis given for one day, (*p* = 0.001).

In the surgical units, 65 HAIs were identified for 11% of patients (62/540). Of these, 56% (35/62) were surgical site infections (35/62) and 95.2% (59/62) were on antibiotics.

About 4 % (14/382) of patients on antibiotics had microbiological analysis done on at least one clinical sample: eight of these in three district hospitals, none of which had microbiological services, but in such cases sent samples to private laboratories, and six from one teaching hospital.

## Discussion

This study shows a high prevalence of antibiotic use (70.7%) among patients admitted to surgical wards across selected hospitals in Ghana. The study population represents almost a 1/3 of the hospital capacity in Ghana and is a fair representation of the surgical population across the country. Although teaching hospitals have a larger proportion of surgical patients due to their specialized personnel/ services and their overall bed size capacity, the results are likely to be representative of the country as a whole.

Antibiotic use was highest in patients admitted with urological conditions, and amongst patients who had undergone a surgical procedure or had developed a HAI. All the patients admitted with a urologic condition in this study were from a single tertiary hospital where there is a long waiting time for patients to see urologists and a longer time still for those requiring surgeries to have them done. Such patients may develop recurrent urinary tract infections requiring antibiotics or if surgery is performed on the urinary system, have an increased risk of developing SSIs. This may explain the higher use of antibiotics. There are low numbers of urologist in the country and lack of a urology center dedicated to managing such large numbers of patients.

Interestingly, the primary indication for antibiotic use was treatment of community-acquired infections, whereas only 1/3 was used for surgical prophylaxis. This calls into question the usefulness of previous studies in surgical wards in LMIC that have mostly focused on surgical prophylaxis [16–19].

The rate of antibiotic use in this study is similar to the 70.8% recorded over a decade ago in a teaching hospital in Ethiopia [20] and higher than the 51% recorded in a previous study looking at antibiotic use overall in a tertiary facility in Ghana [21]. The prevalence is higher than what is reported from the United States, 50% [22] and in acute care hospitals in Europe, 36% [15]. The high rate of antibiotic use brings to fore the absence of a national antibiotic use policy at the time of the study that would guide and control the use of antibiotics in Ghana [23]. The policy on antimicrobial use and resistance for Ghana [24] and the Ghana national action plan on antimicrobial resistance [25], both documents launched in 2018 by the Ministry of Health, Ghana, may be important to counteract the lack of antimicrobial resistance surveillance for both humans and animals [12]. The new guidelines may address, with their aim to improve infection control, pharmacovigilance and post-marketing surveillance of medications as well as expanding laboratory testing, the concern of high rates of antibiotic consumption demonstrated by this study.

There was a high use of 2nd and 3rd generation cephalosporins in community- and hospital-acquired infections as well as for medical and surgical prophylaxis. This goes against the recommendations in the standard treatment guideline of the Ministry of Health of Ghana to avoid cephalosporins to help reduce development of antibiotic resistance in the country [26]. In contrast, the guidelines recommend the use of penicillins (amoxicillin, ampicillin or cloxacillin) for community-acquired infections [26]. It is likely that clinicians wished to safeguard patients by using broader spectrum antibiotics [27]. In the absence of microbiological diagnosis, there was no possibility of scaling down to the appropriate antibiotic therapy based on results of culture and antibiotic susceptibility tests [28]. On average, 1.6 antibiotic agents were administered per patient. In one European study, 1.36 antibiotic agents are prescribed per patient on antibiotics [15]. Potentially, the number of antibiotics per prescription could also be reduced if microbiological analysis was carried out to find the most appropriate therapy. Even in the absence of microbiological testing at the individual level, regular microbiological surveys, including sensitivity testing, would be useful to inform treatment guidelines.

In this study, the most commonly used antimicrobials were metronidazole, cephalosporins, amoxicillin/clavulanic acid, fluoroquinolones and clindamycin, and similar agents were used for antibiotic therapy for community- and hospital-acquired infections as well as for surgical prophylaxis. Carbapenems were used at a low rate and only in tertiary hospitals. Our study design did not allow for us to directly judge the appropriateness of the choice of antibiotics. We did, however, notice that half of the antibiotics given for surgical prophylaxis were nitroimidazole and cefuroxime as recommended in the Ghana standard treatment guideline for use as surgical antibiotic prophylaxis, especially in the instances of colorectal or appendix surgery [26].

Most patients, having spent a median of nine days in hospital prior to the survey, would have had ample time for microbiological analysis to be carried out. The low rate of routine microbiological testing has been attributed to lack of diagnostic facilities [23, 29]. Regional and tertiary institutions in Ghana are all equipped with diagnostic facilities including the 10 study sites. However, only half of the study sites had facilities for microbiological analysis. The low rate of microbiological testing may not only be due to lack of microbiological testing facilities as seen in this study where district hospitals that did not have microbiological services used the services of private laboratories. Clinicians are key in the fight against antimicrobial resistance and their education on the need for microbiological testing whilst prescribing empirical antibiotics is important. Another reason for the low rate of microbiological testing may be that microbiological analyses are not covered by the National Health Insurance Scheme (NHIS) in Ghana. Thus, the patients will have to bear these costs [30]. The NHIS in Ghana, with an almost 40% coverage of the population [31], would have to review its cost covering policy to help reduce this low rate of microbiological testing. The lack of qualified microbiologists, unstable supplies of culture media and other essential reagents and equipment, as well as lack of tradition to do cultures may also account for the low rate of microbiological testing [32].

Previous antimicrobial treatment and excessive duration of treatment are the two most important factors in the selection of resistant microorganisms [33]. Though this study did not look at previous use of antibiotics in the admitted patients, it found that the median duration of administration of antibiotics on the survey date, across selected hospitals was five days, with some antibiotics administered for as long as 89 days. Surgical prophylaxis was generally given for long durations, i.e., more than the one day that is evidence-based and recommended by the World Health Organization [34]. Prolonged surgical prophylaxis does not reduce the number of wound infections and is rather associated with an increased risk of antimicrobial resistance and side effects [35].

Our study shows a widespread need for antibiotic stewardship programs, and for qualitative and quantitative monitoring and evaluation of healthcare professionals’ attitudes toward antibiotic use and microbiological testing. It also suggests the need for improving microbiological services, to forestall the problem of inadequate antibiotic use [36–38], with clinical microbiologist playing a key role in antibiotic stewardship programs. Currently, there is limited evidence for the effect of antibiotic stewardship programs in LMIC. Our findings provide a baseline, urgently needed for studies that may provide such evidence.

### Limitations

The data were collected based on a review of the health records, i.e., paper records of patients who were on admission on the survey day. The district and regional hospitals used similar booklets for everyday recording of patient management and teaching hospitals used similar folders. Because there was limited variation in the format of the records, both types of paper records were easy to review and deemed reliable for obtaining standard information on patient management.

The data on antibiotic use were limited, however, by the fact that this report only looks at the antibiotic use on the day of the survey and not all the antibiotics used by patients during the current admission, prior to the survey date. In addition, all indications were based on the judgement of the attending clinicians and were not scrutinized by the research team.

The timing of administration of surgical antibiotic prophylaxis was not assessed in the study.

The microbiological analysis may be underreported since only available microbiological reports were reviewed without respect to those which had been requested or those done and awaiting results, and this represents another limitation.

## Conclusion

There is a high prevalence of antibiotic use in surgical practice at all levels of hospital care throughout Ghana. Our study elucidated problems on the appropriate use of antibiotics, which included long duration of antibiotic use and limited microbiological testing. For hospitalized patients there is strong evidence from high income countries of an effect of antibiotic stewardship programs on reduction of antibiotic use and resulting benefits at individual and societal levels. Our findings form the basis of and strongly call for studies in Ghana aiming at testing the effects of tailored antibiotic stewardship programs in LMIC. Policy guidelines on antibiotic use should be re-emphasized for all stake holders in Ghana.

## Additional file

Additional file 1: **Table S1**: Combination of antibiotics prescribed in patients. This shows antibiotic combinations prescribed in patients. Each column is the antibiotic group added on to the initial column for the 382 patients. A row gives an indication of antibiotics combination for patients (number of patients on that antibiotic). NB. 169 patients were on 1 antibiotic, 178 patients on 2 antibiotics, 29 patients on 3 antibiotics and 6 patients on 4 antibiotics. (DOCX 18 kb)

## Data Availability

The datasets used and/or analysed during the current study are available from the corresponding author on reasonable request.

## Abbreviations

BSI: Blood stream infection
CAI: Community acquired infection
CI: Confidence interval
ECDC: European Center for Disease Prevention and Control
GIT: Gastrointestinal tract
HAI: Healthcare associated infection
IQR: Interquartile range
KBTH: Korle-Bu Teaching Hospital
PPS: Point Prevalence Study
SP: Surgical prophylaxis
SSI: Surgical site infection
SSTBJ: Skin, soft tissue, bone and joints
UTI: Urinary tract infection
WHO: World Health Organization
