# Supplementary material for: Antibiotic use in surgical units of selected hospitals in Ghana: a multi-centre point prevalence survey
Source: BMC Public Health. 2019 Jun 21;19:797. doi: 10.1186/s12889-019-7162-x (PMC6588883; doi:10.1186/s12889-019-7162-x)
Supplement: Supplementary file 1 — Table S1: Combination of antibiotics prescribed in patients. This shows antibiotic combinations prescribed in patients. Each column is the antibiotic group added on to the initial column for the 382 patients. A row gives an indication of antibiotics combination for patients (number of patients on that antibiotic). NB. 169 patients were on 1 antibiotic, 178 patients on 2 antibiotics, 29 patients on 3 antibiotics and 6 patients on 4 antibiotics. (DOCX 18 kb) [file 12889_2019_7162_MOESM1_ESM.docx]

Supplementary table 1: Combination of antibiotics prescribed in patients

| Antibiotic group 1  (n = 382) | + Antibiotic Group2  (n = 213) | + ANTIBIOTIC Group 3  (n = 35) | + ANTIBIOTIC Group  4 (n = 6) |
| --- | --- | --- | --- |
|  |  |  |  |
| Aminoglycoside (10) | Penicillin (2) |  |  |
|  | Cephalosporin (2) | B-lactam/b-lactamase inhibitor (1) | Lincosamides (1) |
|  |  | Macrolide (1) |  |
|  |  |  |  |
| Penicillin (28) | Nitroimidazole (13) | Cephalosporin (2) |  |
|  | Penicillin (2) |  |  |
|  | Cephalosporin (1) |  |  |
|  | B-lactam/b-lactamase inhibitor (1) | Aminoglycoside (1) |  |
|  | Lincosamides (1) |  |  |
|  |  |  |  |
| Cephalosporin (92) | Nitroimidazole (22) | Lincosamides (1) |  |
|  | Penicillin (7) | Nitroimidazole (2) |  |
|  | Lincosamides (4) |  |  |
|  | B-lactam/b-lactamase inhibitor (2) |  |  |
|  | Aminoglycoside (2) | Nitroimidazole (1) |  |
|  | Carbapenem (1) |  |  |
|  |  |  |  |
| Carbapenem (5) |  |  |  |
|  |  |  |  |
| B-lactam/b-lactamase inhibitor (88) | Nitroimidazole (35) | Aminoglycoside (2) | Penicillin (1) |
|  |  |  | Cephalosporin (1) |
|  | Cephalosporin (3) |  |  |
|  | Macrolide (2) |  |  |
|  | Lincosamides (1) |  |  |
|  | B-lactam/b-lactamase inhibitor (1) | Nitroimidazole (1) | Nitroimidazole (1) |
|  | Aminoglycoside (1) | Macrolide (1) |  |
|  |  |  |  |
| Lincosamide (51) | Fluoroquinolone (16) | Nitroimidazole (1) |  |
|  | Nitroimidazole (5) |  |  |
|  | Cephalosporin (6) |  |  |
|  | Penicillin (3) |  |  |
|  | B-lactam/b-lactamase inhibitor (1) |  |  |
|  | Folate inhibitor (1) |  |  |
|  |  |  |  |
| Macrolide (1) |  |  |  |
|  |  |  |  |
| Fluoroquinolones (53) | Nitroimidazole (29) | Aminoglycoside (1) | Penicillin (1) |
|  |  | B-lactam/b-lactamase inhibitor (1) |  |
|  | Lincosamides (4) | Aminoglycoside (1) |  |
|  |  | Tetracycline (1) |  |
|  | Nitrofurantoin (3) |  |  |
|  | B-lactam/b-lactamase inhibitor (3) | Nitroimidazole (2) |  |
|  | Cephalosporin (1) |  |  |
|  |  |  |  |
| Nitroimidazole (51) | Cephalosporin (16) | Fluoroquinolones (3) |  |
|  |  | Aminoglycoside (3) |  |
|  |  | Cephalosporin (1) |  |
|  | B-lactam/b-lactamase inhibitor (7) | Aminoglycoside (1) |  |
|  | Lincosamides (2) | Cephalosporin (1) |  |
|  | Penicillin (6) | Cephalosporin (1) |  |
|  | Fluoroquinolones (6) | B-lactam/b-lactamase inhibitor (1) | Macrolide (1) |
|  | Aminoglycoside (1) | Cephalosporin (1) |  |
|  |  |  |  |
| Nitrofurantoin (2) |  |  |  |
|  |  |  |  |
| Folate inhibitor (1) |  |  |  |
